# Gankyrin gene deletion followed by proteomic analysis: insight into the roles of Gankyrin in Tumorigenesis and Metastasis

**DOI:** 10.1186/1755-8794-5-36

**Published:** 2012-08-22

**Authors:** Xue Luo, Liang Chen, Jiang Dai, Yanfei Gao, Hongli Wang, Na Wang, Yongqiang Zhao, Feng Liu, Zhihong Sang, Jie Wang, Weihua Li, Kun He, Baofeng Jin, Jianghong Man, Wei Zhang, Qing Xia

**Affiliations:** 1 National Center of Biomedical Analysis, 27 Taiping Road, Beijing, 100850, China; 2 Department of Occupational Health, Third Military Medical University, 30 Gaotanyan Road, Chongqing, 400038, China; 3 Department of Clinical Laboratory, No. 307 Hospital, Academy of Military Medical Sciences, Beijing, 100071, China

## Abstract

**Background:**

Gankyrin was originally purified and characterized as the p28 component of the 26S proteasome, and later identified as an oncogenic protein in hepatocellular carcinomas (HCC). It has recently been found to be highly expressed in several other malignancies, and compelling evidence show gankyrin plays important roles in tumorigenesis. However, its mechanism of action remains unclear.

**Methods:**

In order to further clarify the functions of gankyrin and better understand its molecular mechanisms, we generated a gankyrin null cell line, HCT116 gankyrin^−/−^ , by targeted homologous recombination in human colon cancer cells, and then employed two-dimensional electrophoresis (2-DE) based proteomic approaches followed by MS identification to investigate alterations in the proteome due to the gankyrin knockout. Western blot and qRT-PCR assays were also used to examine the protein and mRNA levels of some identified proteins.

**Results:**

Compared with wild-type control cells, gankyrin null cells were impaired in terms of their proliferation, migration and anchorage-independent growth. A total of 21 altered proteins were identified, which included 18 proteins that had not previously been reported to be related to gankyrin. Notably, eight metastasis-related proteins were identified. Western blot analyses confirmed that the changes in three examined proteins were consistent with 2-DE gel analysis.

**Conclusions:**

In summary, we have generated a useful cell tool to clarify the functions of gankyrin. Our proteomic data provide novel information to better understand the roles and underlying mechanisms by which gankyrin is involved in tumorigenesis and cancer metastasis.

## Background

Gankyrin was originally purified and characterized as the p28 component of the 19S regulatory subunit of the 26S proteasome. It consists of 226 amino acids that encode a 25-kDa protein with six ankyrin repeats [1]. Fujita et al. identified gankyrin as an oncogenic protein that is overexpressed in hepatocellular carcinomas (HCC) [2]. They and several other groups have demonstrated critical roles of gankyrin in HCC tumorigenesis [2-4]. Gankyrin has recently been found to be highly expressed in several other malignancies, including lung cancer [5], pancreatic cancer [6] and colorectal cancer [7]. Compelling evidences from the literature and our own findings show that gankyrin plays important roles in both tumorigenesis and metastasis.

We and other investigators have been devoted to unraveling the mechanism that underlies the oncogenic effects of gankyrin. [Hiroaki H et al. 4] found that gankyrin controlled two major tumor suppressors, Rb and p53. Previously, we observed that gankyrin was upregulated in Ras-transformed cells [8], and it played an essential role in Ras-initiated transformation by regulating Akt activation [5]. However, its detailed functions and mechanism of action as a whole remain unclear.

In order to further clarify the functions of gankyrin and better understand its molecular mechanisms, we generated a gankyrin null cell line from the human colon cancer cell line, HCT116, by gene targeting. The utilization of HCT116 as the gene targeting cells was borne out of two considerations: Firstly, [Tang et al. 7] reported that gankyrin is significantly overexpressed in colorectal cancer tissues and cell lines. We tested the protein expression levels of gankyrin in 50 paired samples of colorectal cancer and adjacent normal tissue, and obtained similar results (unpublished data). Secondly, with a stable chromosomal complement, HCT116 as a diploid cancer cell line has been utilized most often for the generation of knockout and knock-in mutations [9]. Previous studies regarding gankyrin have mainly been based on RNA interference (RNAi) or overexpression experiments. However, RNAi might generate off-target effects that make its results difficult to predict and interpret. On the contrary, gene targeting by homologous recombination is a powerful technique for the analysis of gene function, which produces results that are more straightforward to interpret than overexpression or RNAi studies [10]. Specifically, human somatic cell knockout is a unique model system to study human gene function with conceptual advantages over analogous studies in model organisms. Based on similar strategies, several human knockout cell lines have been established and widely used, such as HCT116 p53^−/−^, HCT116 PPARð^−/−^ and HCT116 CDC4^−/−^ cells, amongst others [11-15].

In the current study, we established a knockout cell line, HCT116 gankyrin^−/−^, which was a unique and powerful tool to understand the biological functions of gankyrin. We also employed two-dimensional electrophoresis (2-DE) based proteomic approaches followed by MS identification to investigate alterations in the proteome due to the gankyrin knockout. A total of 21 altered proteins were identified, including five that were upregulated and 16 that were downregulated, and these were found to be involved in a variety of cellular functions. Eighteen have not been previously described to be related to gankyrin. A notable finding was the identification of eight cancer metastasis-related proteins. Our data paves the way to a better understanding of the mechanisms by which gankyrin is involved in tumorigenesis and cancer metastasis.

## Methods

### Plasmids, cells, transfection and reagents

The gene targeting vector, pAAV-puro, was a gift of Dr. Guang bin Luo (Case Western Reserve University, USA). HCT116 (American Type Culture Collection, Rockville, MD, USA) and its derivatives were grown in 10% FBS and 1% penicillin-streptomycin in McCoy’s 5A modified media and maintained at 37°C in 5% CO_2_. Cells were transfected with Lipofectamine 2000 (Invitrogen, Carlsbad, CA, USA), following the manufacturer’s protocol and colonies were selected with the use of 1 μg/ml puromycin. The anti-p53 (sc-126) and anti-gankyrin (sc-8991) antibodies were purchased from Santa Cruz Biotechnology, Inc. (Santa Cruz, CA), and the antibody against PIG3 (BS2085) and ANAX2 (BS3553) were purchased from Bioworld Technology, Inc. (Bioworld, USA). The monoclonal anti-α-tubulin (T5168) was purchased from Sigma.

### Generation of gankyrin null cells

The general strategy for creating the gankyrin null line was described by [Bunz et al. (2007) 9]. Briefly, the 5’ and 3’ homology arms used for constructing the targeting vectors were PCR-amplified from HCT116 genomic DNA, using primers chosen from publicly available genomic sequence databases (Gene ID: 5716). The arms were cloned into vectors that contained a hygromycinythymidine kinase fusion gene that was flanked by LoxP sequences. The primers used to derive the targeting vectors and details of their construction are available from the authors upon request. Screening for homologous recombination events was performed by PCR (forward: 5’-ATGTATTCTTATCGTTACCTAGT-3’; reverse 5’-GGACGTAAACTCCTCTTCAGA-3’). After Cre-mediated LoxP excision, allele-specific primers were used for further genetic verification. The above forward primer was used with the following reverse primers: 5’-CTGTTTTGACTGGCGTAGCC-3’ for the wild-type allele and 5’-TTCTGCTTCTCTCAGAAACGG -3’ for the deleted allele. Lox recombination was mediated by transfecting the cells with a Cre expressing plasmid, pCX-Cre. All targeted clones identified by PCR were verified by Southern blotting with 10 mg of genomic DNA digested with the restriction enzyme *Hind*III and *Aat*II, and then probed with a 600-bp genomic fragment lying inside the 5’ homology arm.

### Cell growth curve

Equal numbers (2 × 10^4^) of HCT116 gankyrin^−/−^ cells or wide type cells were plated into 12-well tissue culture dishes and cell numbers were determined by counting with a hemocytometer at 0, 1, 2, 3, 4, 5 and 6 days of cell plating. Each assay was completed in triplicate.

### Soft agar assay and tumorigenicity in nude mice

To assess the anchorage independency of growth, 5 × 10^3^ cells were plated in 0.6% agar layered on top of 1.0% agar in 6-well plates, and colonies were counted after 2~3 weeks of incubation at 37°C and 5% CO_2_ in air. Each assay was completed in triplicate. Statistical differences between the sample means were calculated by analysis of variance (ANOVA), followed by an unpaired Student’s t-test. The results are expressed as the mean ± standard error of the mean (SEM). The animals used in this study were nude mice, 4~6 weeks old (National Center of Biomedical Analysis, Beijing). HCT116 gankyrin^−/−^ cells and control cells (5 × 10^6^) were implanted by subcutaneous injection into the dorsal region near the thigh of female nude mice. Tumor volume was measured at the indicated days.

### Cell migration assay

The cell migration assay was performed using Oris^TM^ Cell seeding stoppers (Platypus technologies, Madison, WI) according to the manufacturer’s protocol. Briefly, the assay utilizes Oris^TM^ Cell seeding stoppers (made from a medical-grade silicone) to restrict cell seeding to the outer annular regions of the wells. Cells were seeded onto each well (5 × 10^4^ cells/well) and allowed to attach for 4 h at 37°C. The stopper was subsequently removed to form an unseeded region (2 mm in diameter) at the center of each well. The plate was incubated at 37°C to permit cell migration, and the migrated cells were stained with crystal purple.

### Protein preparation

To obtain total protein lysates, 80~90% of confluent cells was washed with chilled phosphate buffered saline (PBS), and cell lysates were then prepared on ice using cool lysis buffer (8 M urea, 4% CHAPS, 40 mM Tris, 1 mM EDTA, 1 mM EGTA, 60 mM DTT) containing a protease inhibitor cocktail (Roche Diagnostics, Mannheim, Germany). The sample was aliquoted and stored at −70°C until use.

### 2D electrophoresis and image analysis

Proteins were separated by 2-DE as described previously [16]. Briefly, isoelectric focusing was performed with the IPGphor system (Amersham Pharmacia Biotech, Uppsala, Sweden). Several different IPG (immobilized pH gradient) strips (18 cm, pH 3~10 nonlinear, 3~5.6, 6~11, Bio-Rad Co.) were used. After active rehydration for 12 h at 30 V, the strips (18 cm, pH 3~10, nonlinear) were focused at 0.05 mA/IPG strip for 80,000 Vh at 20°C. Once the IEF was finished, the IPG strips were immediately equilibrated in 10 ml of equilibration solution (6 M urea, 30% glycerol, 2% sodium dodecyl sulfate [SDS], 50 mM Tris-Cl pH 8.8, 1% dithiothreitol [DTT]) with gentle shaking for 15 min. The strips were then treated with the same solution containing 2.5% iodoacetamide instead of DTT. SDS-PAGE was performed using 13% polyacrylamide gels without a stacking gel in the PROTEAN II cell (Bio-Rad Co.). Following SDS-PAGE, gels were stained with 0.1% (w/v) Coomassie Blue G-250 (CBB G250) in 50% methanol and 10% acetic acid, or silver-stained. Spot detection, quantification, and matching were performed with Image Master 2-D Elite Version 5.0 software according to the manufacturer’s instructions (GE Healthcare Life Sciences, Uppsala, Sweden). Proteins were subjected to further analyses when the expression levels of given protein spots changed by at least 1.5-fold. Each experiment was performed at least in triplicate.

### Protein identification

2-DE gels were stained with silver or CBB G250. The protein spots were excised from the 2-DE gels and in-gel digested as described previously. Protein identification was repeated at least twice using spots from different gels. Protein spots were analyzed by NanoLC-HDMS MS/MS on an AcquityTM Nano UPLC system (Waters Corp., Milford, USA) and Synapt high-definition mass spectrometry (HDMS) was performed with a nanospray ion source (Waters). The HDMS was operated in a data-dependent mode with MS/MS scans (2 seconds). The voltage of non-coated capillary was set as 2300 V.

Glufibrinopeptide was used to calibrate the instrument in the MS/MS mode. Peak lists were generated using PLGS 2.2 software and automatically combined into a single pkl file for every LC-MS/MS run. The MS/MS data were acquired and processed using MassLynx V4.1 software (Micromass) and Mascot from Matrix Science in March 2011 was used to search the database using the following parameters: database, NCBInr (13366630 sequences); taxonomy, *Homo sapiens* (235802 sequences); enzyme, trypsin; and one missed cleavage was allowed. Carbamidomethylation was selected as a fixed modification and oxidation/phosphorylation was allowed to be variable. The peptide and fragment mass tolerances were set at 1 and 0.2 Da, respectively. Proteins with probability-based MOWSE scores that exceeded their threshold ( *p* < 0.05) were considered to be positively identified. If proteins were identified by a single peptide, the spectrum was validated manually. For a protein to be accepted, the assignment had to be based on four or more y- or b-series ions.

### qRT-PCR

Total RNAs were isolated from 1 × 10^6^ cells with TRIzol reagent (15596–026, Invitrogen) according to the manufacturer’s instructions. Primers used to amplify the gene fragments are available from the authors. MMLV Reverse Transcriptase (M1701, Promega, Madison, WI, USA) were used for cDNA synthesis. Transcripts were quantified by qRT-PCR on an ABI PRISM 7300 Sequence Detector (Perkin-Elmer Applied Biosystems) with TaKaRa predisigned SYBR® Premix Ex Taq™ assays and reagents (TaKaRa, Dalian, China) according to the manufacturer’s instructions. The comparative threshold cycle method and an internal control (GAPDH) were used to normalize the expression of the target genes.

## Results

### Generation of gankyrin null HCT116 cells

**Figure 1:**
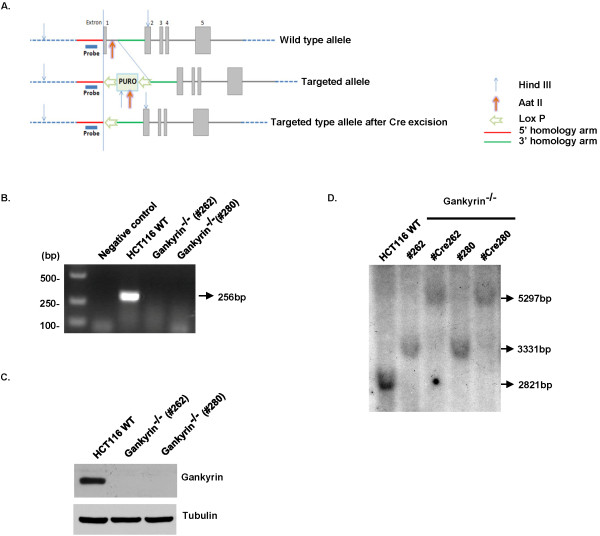
**Knockout of gankyrin by homologous recombination.** ( **A**) Constructs used to mediate homologous recombination at the gankyrin locus. Exon 1, containing the ATG start codon, was targeted for deletion by homologous recombination using 5’ and 3’ homology arms that were 957 bp and 902 bp in length, respectively. The wild-type allele, targeted allele, and targeted allele after Cre-mediated excision are illustrated. ( **B**) Confirmation of the knockouts by a PCR screen of genomic DNA. ( **C**) Southern blot analysis confirmed DNAs were digested with *Hind*III and *Aat*II and hybridized with the probes depicted in ( **D**). ( **E**) Western blot analysis confirmed the protein expression levels of gankyrin. Western blot for tubulin was also carried out to confirm equal loading.

To explore the function of gankyrin in human cancer cells in a rigorous manner, we chose to disrupt the endogenous gene by targeted homologous recombination in a human colorectal cancer cell line. A recombinant adeno-associated virus (rAAV) mediated gene targeting vector was designed so that exon 1 was disrupted by promoterless antibiotic-resistance genes (Figure 1A). As the gankyrin gene is located on the X chromosome, only a single round of rAAV-mediated recombination was performed. We obtained two independent gankyrin^−/−^ clones (#262, #280) from 400 neomycin-resistant colonies according to a PCR screen of genomic DNA (Figure 1B). The expression of the gankyrin protein in the two knockout clones was demonstrated by Western blot analysis with an anti-gankyrin antibody (Figure 1C). The correct targeting events were further confirmed by Southern blot analysis (Figure 1D).

### Gankyrin deletion decreased colon cancer cell growth rate and migration

**Figure 2:**
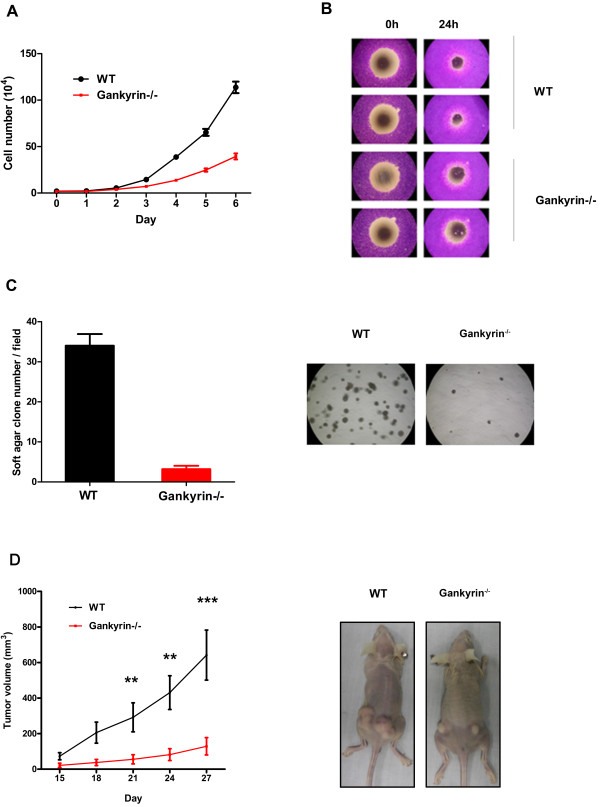
**Cell proliferation, anchorage-independent growth and migration abilities were compromised by the gankyrin knockout.** ( **A**) Growth curves, showing equal numbers of indicated cells (2 × 10^4^) were plated into 12-well tissue culture dishes. Cell numbers were counted at 0, 1, 2, 3, 4, 5 and 6 days. Triplicate wells were used for each time point. The results show the means ± the standard error of the mean for three independent experiments. (**B**) Cell migration analysis. Cells were seeded onto Oris collagen I-coated plates populated with Oris cell seeding stoppers. After 48 h, the cells were fixed and stained with crystal violet. ( **C**) Analysis of the levels of cell growth in soft agar. Cells were mixed with soft agar and seeded into 6-well plates; the number of foci was then determined 2~3 weeks later. Data are expressed as the total number of colonies per plate. ( **D**) Nude mice tumor growth assay. Nude mice (n = 5) were injected subcutaneously in each flank with 5 × 10^6^ cells, and tumor growth was monitored for 3~4 weeks by caliper measurements. Data are shown as the means ± SEM.

Figure 2A compares the growth curves of HCT116 gankyrin^−/−^ and wild-type cells. We calculated the rate of growth of the cell lines by counting the total number of cells in duplicate wells every day for up to 6 days. A marked inhibition of cell growth was observed in gankyrin^−/−^ cells. We also performed cell migration assays with Oris^TM^ Cell seeding stoppers. The number of gankyrin^−/−^ cells that migrated into the unseeded region was significantly decreased compared with wild-type cells (Figure 2B), which demonstrates the role of gankyrin in cell migration and the metastasis of colorectal cancer.

### Gankyrin is required for efficient tumorigenicity

We have previously reported that gankyrin was upregulated in Ras-transformed NIH3T3 cells. When gankyrin expression was knocked down in Ras G12V cells, fewer colonies formed in soft agar. Consistent with these previous effects, the ability of HCT116 gankyrin^−/−^ cells to form colonies in soft agar was significantly impaired in this present study (Figure 2C). The anchorage-independent proliferation assay results further confirm the oncogenic activity of gankyrin in transformation. To investigate the effect of gankyrin on tumorigenicity in vivo, wild-type HCT116 and gankyrin^−/−^ cells were injected as xenografts in nude mice. There was a dramatic difference in tumor growth of the gankyrin^−/−^ cells compared with wild-type HCT116 cells (Figure 2D).

### Two-dimensional proteome maps to identify changes in protein expression as a result of the gankyrin knockout

**Figure 3:**
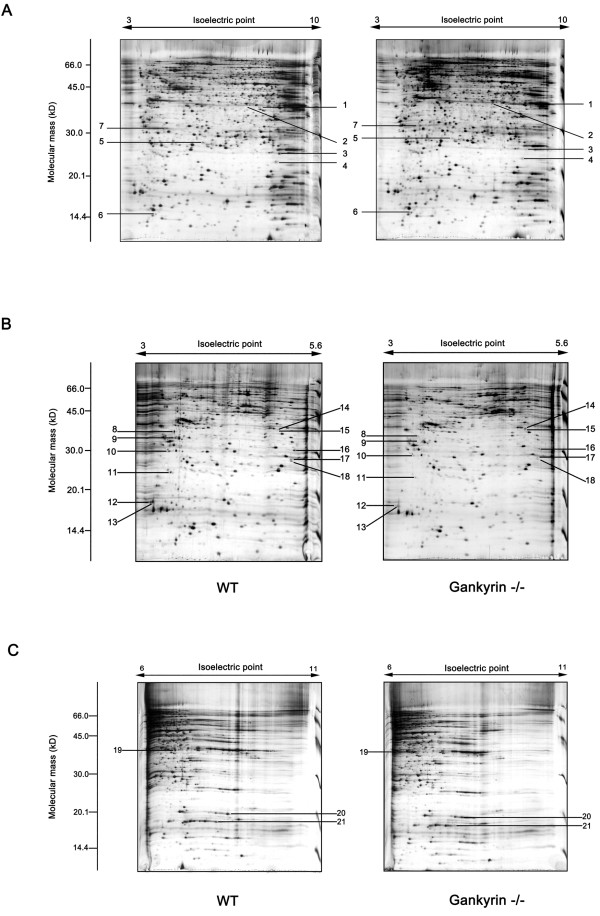
**2-DE analysis of differentially expressed protein spots between HCT116 gankyrin**^**−/−**^**and wild-type cells.** Total cell lysates (125 μg) of each cell lines were subjected to 2-DE analysis and detected by silver staining. The pH gradient of the first-dimension electrophoresis (3~10, 3~5.6, 6~11) is shown on top of the gels, and the migration of molecular mass markers for SDS-PAGE in 2-D is shown on the left side. The original gel size was 20 × 20 × 0.1 cm^3^. The marked protein spots were significantly altered between HCT116 gankyrin^−/−^ and wild-type cells. The results of identification by mass spectrometry are listed in Table 1.

**Figure 4:**
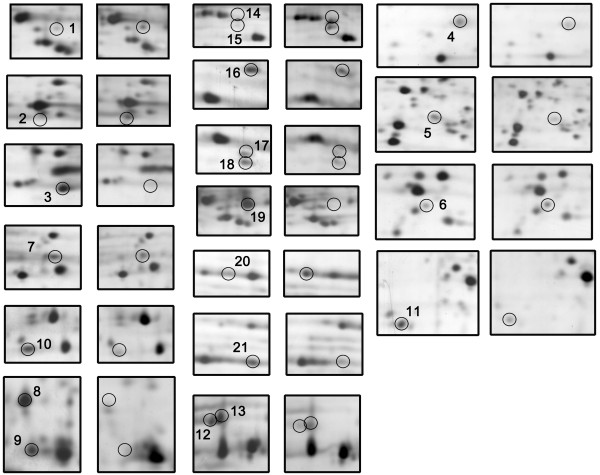
**Close-up sections of altered protein spots between HCT116 gankyrin**^**−/−**^**and wild-type cells.** The sections compared are the same as those for silver-stained 2-DE gels shown in Figure 3. The number of protein spots is shown in Table 1.

Cell lysates from the two cell lines were resolved by 2-DE. All of the spots marked in Figure 3A were distributed in *pI* 3~10 and their molecular masses ranged from 14 to 116 kDa. In order to gain a better resolution, narrower *pI* gradients IPG gels were used, including *pI* 3~5.6 (Figure 3B) and *pI* 6~11 (Figure 3C). We reproducibly detected more than 1,000 protein spots on 2-D gels after silver staining. Each 2D map was repeated at least five times. From the gels, we detected 21 differentially expressed protein spots. Among the spots marked in Figure 3, five proteins were upregulated, while 16 were downregulated due to the gankyrin knockout (Table 1). The close-up section patterns of the 21 altered protein spots, shown in Figure 4, clearly indicated the expression level changes, whereas certain other spots within the same section were unchanged.

**Table 1:** **List of proteins identified by mass spectrometry significantly changed between HCT116 gankyrin**^**−/−**^**and HCT116 cells**

**Spot No**	**Protein name**	**NCBI ID No.**	**Abbr nme**	**Mr (kD)**		***pl***		**Sequence Coverage (%)**	**Score**	**Change Fold**
				**Thero**	**Ober**	**Thero**	**Ober**			
1	Tumor protenin P53 Inducible protein 3	AAC39528	Pig3	34.4	40.2	5.9	6.0	10	115	+1.9
2	Cytochrome P450 family 1subfamily A	AAX62803	CYP1A1	54.7	39.1	8.3	7.2	2	41	+3.2
3	Phosphatidylethanolamine binding protein	AAB32876	PEBP1	20.9	24.9	7.4	8.0	50	347	-5.6
4	NEDD8-conjugating enzyme Ubc12	NP_003960	Ubc12	20.8	24.1	7.7	7.9	42	196	-2.0
5	26S proteasome non-ATPase regulatory subunit 10 isoform 1	NP_002805	Gankyrin	24.4	28.8	5.7	5.9	29	30	-3.3
6	S100-A9	NP_002956	S100-A9	13.2	15.0	7.4	4.2	33	149	+2.1
7	Protein kinase C inhibitor protein 1	AAH51814	YWHAZ	35.3	30.1	4.5	4.2	39	347	-9.8
8	Proliferating cell nuclear antigen	1AXC_C	PCNA	28.7	38.2	4.4	3.8	44	229	-9.1
9	Tropomyosin alpha-4 chain isoform 2	NP_003281	TPM4	28.5	34.9	4.5	3.8	40	274	-3.6
10	Eukaryotic translation factor 6 isoform a	NP_002203	p27BBP	26.5	30.1	4.4	3.7	15	131	-2.2
11	Nucleoplasmin-3	NP_008924	NPM3	19.3	27.0	4.4	3.7	14	182	-3.0
12	Peptidyl prolyl cis trans isomerase A	NP_066953	PPIA	17.8	17.5	7.7	3.4	33	54	-5.0
13	Calcium/calmodulin-dependent protein kinase II gamma	1J7O_A	CAMK2G	8.4	17.5	3.79	3.48	39	107	-2.9
14	L-lactate dehydrogenase B chain	NP_002291	LDHB	36.6	45.3	5.6	5.2	8	96	+5.2
15	G protein beta subunit	AAA35922	GNB2	37.3	45.1	5.7	5.2	23	218	+6.3
16	Proteasome subunit beta type-7	NP_002790	PSMB7	29.9	30.1	7.6	5.5	5	91	-26
17	Prosome beta subunit	AAB31085	PSME2	25.8	29.8	5.6	5.4	28	175	-1.6
18	Proteasome subunit beta type-4	NP_002787	PSMB4	29.2	29.6	5.6	5.4	28	188	-2.3
19	Annexin A2 isoform 2	NP_004030	ANXA2	38.6	38.2	7.8	7.1	51	366	-10.5
20	Cofilin-1	NP_005498	Cofilin-1	18.5	18.9	8.2	8.4	50	347	-2.6
21	Chain K, Acetyl-Cypa:cyclosporine Comples	2X2C_K	PPIA	18.0	18.0	7.2	8.1	89	1473	-3.0

The 21 protein spots were subjected to in-gel trypsin digestion and analysis with ESI-MS/MS. We successfully identified all 21 protein spots (Table 1). Protein spot 5 that disappeared in HCT116 gankyrin^−/−^ cells was identified as gankyrin. The identification of gankyrin only in wild-type cells confirmed the successful establishment of the gankyrin knockout cell line, as well as the reliability of the 2DE/MS strategy. In our results, some identified proteins showed *pI* and Mr shifts from the theoretical values, such as protein cytochrome P450, family 1, subfamily A, polypeptide 1 (CYP1A1) for spot 2, and S100 calcium binding protein A9 (S100-A9) for spot 7 (Table 1). Changes in *pI* and Mr are often attributed to post-translational modifications of proteins, such as glycosylation, phosphorylation and protease digestion, which are usually required for proteins to carry out their biological functions. These differentially expressed proteins are involved in different cellular functions, including protein degradation, cell motion, stress and defense, cell cycle control and metabolism (Table 2). Among them, eighteen proteins have not been reported previously to be connected with gankyrin. Notably, eight metastasis-related proteins were found to be regulated by gankyrin. The identification of these gankyrin-regulated proteins suggests novel functions of this oncogene, and their relevance in oncogenesis and metastasis need to be explored further.

**Table 2:** **Functional clustering of proteins differentially expressed between HCT116 gankyrin**^**−/−**^**and HCT116 cells**

**Functional clustering**	**Protein name**
Protein degradation	PSME2, PSMB4, PSMB7, Ubc12
Cell motility	Cofilin 1, ANXA2, YWHAZ, S100A9, TPM4, PPIA, PEBP1, p27BBP
Stress and defense	PIG3, PPIA, CAMK2G
Cell cycle control	CYP1A1, PCNA, Ubc12
Metabolism	LDHB, GNB2, CYP1A1
Unclassified	NPM3, AC2C_K

### qRT-PCR and Western blot analysis

**Figure 5:**
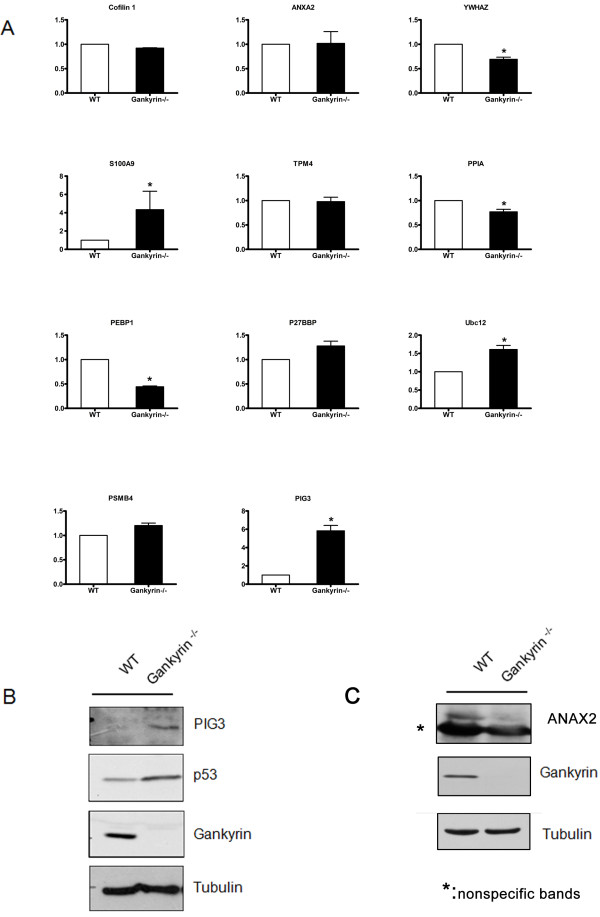
**Real-time quantitative PCR analysis and Western blot confirmation of the differentially expressed proteins.** ( **A**) The mRNA expression levels of 11 differentially expressed proteins in 2-DE gels were analyzed by qRT-PCR. GAPDH was used as the internal control. Upregulation of PIG3, P53 ( **B**) and downregulation of ANAX2 ( **C**) in HCT116 gankyrin^−/−^ cells was confirmed by Western blot analysis.

Those differentially expressed proteins are commonly regulated at the transcriptional level and/or through translational and post-translational modifications. To explore the mechanisms that led to changes in the expression of the identified proteins due to the gankyrin knockout, we selected 11 genes for qRT-PCR analysis, including eight metastasis-related genes and three other genes. For the qRT-PCR analysis, the total RNA isolated from the two cell lines was used as the template. The change at the mRNA level of the four metastasis-related genes (*YWHAZ*, *S100A9*, *PPIA*, *PEBP1*) were consistent with their protein expression levels (Figure 5A); the other four metastasis-related genes were found to have unchanged levels of mRNA. The protein levels of PIG3, ANXA2 and gankyrin were also investigated by Western blot analyses. These results confirmed that the changes in these three proteins were consistent with our 2-DE gel analysis (Figure 5B, 5C).

## Discussion

Although gankyrin has been acknowledged as an important oncogene in HCC and several other malignancies, its oncogenic effects and mechanisms of action remain unclear. In the present study, we generated a human colon cancer cell line, HCT116 gankyrin^−/−^, by gene targeting, which enabled us to rigorously evaluate the role of this oncogene in tumorigenesis and cancer metastasis. By comparing isogenic cell lines that differed only in the presence or absence of the *gankyrin* gene, we have unambiguously shown that this protein can directly affect the growth and migration of colorectal cancer cells.

By 2DE/MS based approaches, we systematically analyzed the alterations in the cellular proteome due to the gankyrin knockout, which enabled the identification of numerous changes in protein expression and post-translational processing. Overall, 21 differentially expressed proteins were identified, including gankyrin and another two proteosome subunits, proteasome subunit beta type 7 (PSMB7) and proteosome beta-subunit 2 (PSME2).

The 26S proteasome is the core machinery of the ubiquitin proteasome system (UPS), which executes the degradation of most unwanted proteins in the cytosol and nucleus. Protein degradation in the proteosome plays an important role in cell proliferation, differentiation, signal transduction and the stress response, amongst others. Defects in the UPS have been linked to many human diseases, including cancer. By affinity purification and tandem mass spectrometry, [Wang et al. 17] characterized the human 26S proteasome complex, and identified gankyrin, PSMB7 and PSME2. These gankyrin–regulated proteins have been reported to play roles in several important biological processes, such as the DNA damage response, cell cycle control, metabolism and cell motility. The dysregulation of these proteins is closely related to tumorigenesis and cancer metastasis.

DNA damage activates a complex signaling network that mediates DNA repair and activates cell-cycle checkpoints. Defects in the ability to properly respond to and repair DNA damage can result in genomic instability and lead to the transformation of normal cells into cancer cells. Three DNA damage response proteins, PIG3, PPIA, CAMK2G, were identified in the present study. PIG3 has been reported to be a p53 downstream target gene, and plays central roles in DNA damage response as a “genome guard”. The observation that PIG3 was upregulated due to the deletion of gankyrin expression was consistent with former reports that gankyrin negatively regulates p53 by increasing its ubiquitynation and degradation. [Hiroaki et al. 18] also reported that, when treated with Adriamycin, a DNA damaging agent, the induction of PIG3 by p53 was decreased by the overexpression of gankyrin. As the RT-PCR results showed that the mRNA level of PIG3 was upregulated in gankyrin knockout cells, we concluded that this resulted from the increasing transcriptional activity of p53.

The dysregulation of proliferation is one of the most fundamental traits of cancer cells. Three cell cycle-related proteins, PCNA, CYP1A1 and Ubc12, were found to be regulated by gankyrin, which sheds light on the molecular mechanisms that underlie the oncogenic activity of gankyrin. PCNA forms a ring around DNA to facilitate and control DNA replication, which plays a role in many other essential cellular processes, such as the maintenance of chromatin structure, chromosome segregation and other cell-cycle progression stages. In this study, PCNA was observed to be dramatically downregulated after the deletion of gankyrin. Yun et al. obtained similar results when gankyrin was knocked down in pancreatic cancer cells.

Ubc12, an E2 NEDD8-conjugation enzyme, is a key molecule in the neddylation cascade. One of its main substrate, cullin, which is part of the SCF ubiquitin E3 ligase complex, plays critical roles in cell cycle progression. This finding suggests that gankyrin could promote cell cycle progression through regulating the neddylation of important cell cycle molecules.

The analysis of clinical samples has shown that gankyrin is overexpressed in colorectal cancer tissues and cell lines compared to controls, and its expression level was correlated with the tumor, node, and metastasis (TNM) staging system [7]. Our wound healing assays demonstrated that the gankyrin knockout significantly decreased cell motility, which provides in vitro evidence of the role of gankyrin in cancer metastasis. Notably, in the present proteomic analysis, 40% of the proteins identified to be involved in cancer metastasis processes, including cofilin 1, annexin A2 isoform 2 (ANXA2), protein kinase C inhibitor protein 1 (YWHAZ), S100A9, chain K acetyl-cypa:cyclosporine complex (PPIA), phosphatidylethanolamine binding protein (PEBP1) and p27BBP. Cofilin is a small ubiquitous protein that can bind both monomeric and filamentous actin [19]. It has been reported to be a key player in regulating the dynamics of the actin cytoskeleton of migrating cells and important for the motility of mammary cancer cells [20]. Cofilin and its regulatory proteins are involved in the initiation of the early steps in the motility cycle, and evidence has emerged that the expression of certain genes of the cofilin pathway are altered in invasive tumor cells. The activity of the cofilin pathway (Rho-Rock-LIMK-Cofilin) is believed as one of the major determinants of metastasis [21]. The inhibition of cofilin in several cell types has been found to alter cell protrusion and motility. [22] In the present study, cofilin was downregulated after the deletion of gankyrin, which was consistent with the phenotype of impaired cell migration. Our previous study revealed that gankyrin is a key mediator of Ras–induced transformation by regulating the RhoA/ROCK/PTEN pathway [5]. Whether gankyrin mediates tumor metastasis by affecting the cofilin pathway needs to be further clarified.

Cyclophilin A (CypA) has peptidylprolyl cis-trans isomerase activity, which plays important roles in protein folding, trafficking, assembly, immune-modulator and cell signaling. The upregulation of CypA in several cancers has been reported, including small cell lung cancer, pancreatic cancer, breast cancer, colorectal cancer and hepatocellular carcinoma (HCC). Zhang et al. reported that CypA promotes HCC cell metastasis through the upregulation of MMP3 and MMP9. Our results suggests that CypA is positively regulated by gankyrin, and CypA-MMP3/MMP9 may underlie the role of gankyrin in cancer metastasis.

ANXA2 is one of the well studied receptors for plasminogen, as it converts plasminogen to plasmin after binding. Plasmin is a serine protease that plays a key role in the activation of metalloproteinases and the degradation of extracellular matrix components that are essential for metastatic progression. Accumulating evidence suggests that ANXA2 and its receptor axis plays an important role in the tumor microenvironment and metastasis, and it has been recognized as an attractive target for the development of anti-cancer/anti-metastatic agents [23,24]. Our 2D/MS result showed that ANXA2 was downregulated due to the gankyrin knockout, which is the first evidence that ANXA2 could be upregulated by gankyrin.

The YWHAZ gene encodes 14-3-3 protein zeta (ζ), a member of the 14-3-3 family, whose members mediate signal transduction by binding to phosphoserine-containing proteins. The upregulated expression of 14-3-3ζ is associated with histological grade, lymph node metastasis and poor clinical outcomes in some cancer types [25]. Recent studies have shown that 14-3-3ζ interacts with many key cellular proteins that are involved in tumor development and progression. [Gohla et al. 26] reported that the 14-3-3ζ protein could regulate cellular actin structures through the maintenance of phosphorylated-cofilin levels. Moreover, 14-3-3ζ forms the regulatory complex with Slingshot-1 L (SSH-1 L), a selective cofilin-regulatory phosphatase. The identification of YWHAZ and other metastasis-related proteins presents novel mechanistic insights into the critical role of gankyrin in cancer metastasis.

## Conclusions

In conclusion, our results demonstrate the utility of rAAV-mediated gene targeting to generate gankyrin null cells, which provides a useful tool to clarify the role of gankyrin in colon cancer tumorigenesis and metastasis. We implemented a proteomics approach for the systematic analysis of cellular proteome changes in HCT116 gankyrin^−/−^ cells. In total, 21 differentially expressed proteins were identified, including 18 proteins previously unknown to be related to gankyrin. These newly identified gankyrin-regulated proteins are involved in various biological functions, including cell cycle control, protein degradation and metabolism, which highlight the versatile functions of gankyrin. However, the present results are based on a single cancer cell line. In view of the heterogenicity of cancer cells, as well as the complexity of their specialized tumor microenvironments in vivo, the functions of gankyrin reported here may not reliably reflect its true roles in diverse human cancers. These functions will need to be confirmed by further in vitro and in vivo experiments.

## Competing interests

The authors declare that they have no competing interests.

## Authors' contributions

XL: performed the 2DE and qRT-PCR analyses and contribute to the manuscript writing. LC: established the gene knock out cells and composed the initial manuscript draft. JD: performed the Western blot analyses. YFG: conducted statistical analysis. HLW, NW, YQZ, FL, ZHS, JW, WHL, KH: performed the mass identification and 2DE image analyses. BFJ and JHM: performed the soft agar analysis and animal experiments. WZ and QX: conceptualized the project and wrote the paper. All authors read and approved the final manuscript

## Pre-publication history

The pre-publication history for this paper can be accessed here:

http://www.biomedcentral.com/1755-8794/5/36/prepub
